# Development of Cationic Lipid LAH4-L1 siRNA Complexes for Focused Ultrasound Enhanced Tumor Uptake

**DOI:** 10.1021/acs.molpharmaceut.2c00909

**Published:** 2023-03-29

**Authors:** Shahd Abuhelal, Miguel N. Centelles, Michael Wright, A. James Mason, Maya Thanou

**Affiliations:** Institute of Pharmaceutical Science, School of Cancer and Pharmaceutical Sciences, King’s College London, Franklin-Wilkins Building, 150 Stamford Street, London SE1 9NH, U.K.

**Keywords:** RNAi, ternary complex, hyperthermia, focused ultrasound, liposomal siRNA delivery

## Abstract

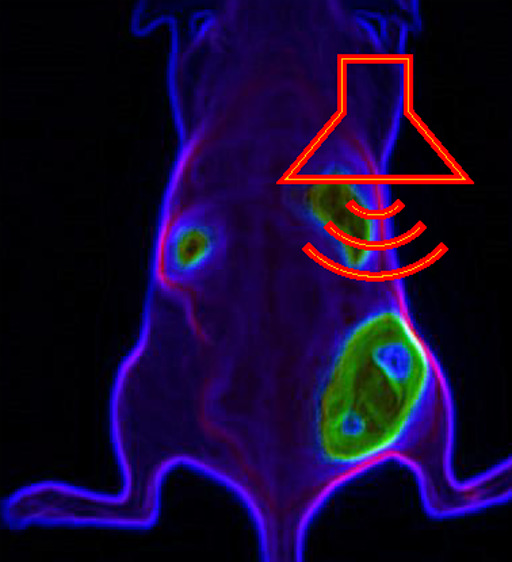

RNAi has considerable potential as a cancer therapeutic approach, but effective and efficient delivery of short interfering RNA (siRNA) to tumors remains a major hurdle. It remains a challenge to prepare a functional siRNA complex, target enough dose to the tumor, and stimulate its internalization into tumor cells and its release to the cytoplasm. Here, we show how these key barriers to siRNA delivery can be overcome with a complex—comprising siRNA, cationic lipids, and pH-responsive peptides—that is suited to tumor uptake enhancement via focused ultrasound (FUS). The complex provides effective nucleic acid encapsulation, nuclease protection, and endosomal escape such that gene silencing in cells is substantially more effective than that obtained with either equivalent lipoplexes or commercial reagents. In mice bearing MDA-MB-231 breast cancer xenografts, both lipid and ternary, lipid:peptide:siRNA complexes, prepared with near-infrared fluorescently labeled siRNA, accumulate in tumors following FUS treatments. Therefore, combining a well-designed lipid:peptide:siRNA complex with FUS tumor treatments is a promising route to achieve robust in vivo gene delivery.

## Introduction

RNA therapeutics have proven to be very important for humankind. Almost everyone globally knows that vaccines for COVID-19 are based on RNA molecules and lipids. However, even before the mRNA vaccines, RNA therapeutics in the form of RNA interference (RNAi) had been used in the clinic for several years in the form of siRNA or RNAi. The first FDA-approved siRNA drug was announced in late 2018 to treat a rare disease, peripheral nerve disease (polyneuropathy) caused by hereditary transthyretin-mediated amyloidosis (hATTR), in adult patients.^1^ This was followed by the approval of Inclisiran, a siRNA drug for the treatment of elevated LDL cholesterol.^2^ RNAi has been considered a promising technology to stop the expression of various oncogenes for treating cancer. The primary limitation of siRNA for clinical application is the safe and efficacious delivery of therapeutic siRNA into the tumor and cancer cells to effect on the genetic target. However, although extensive studies^3^ have shown its use,^4^ no siRNA has yet been approved for cancer treatment.^5^

The goal of siRNA therapy is to achieve therapeutic activity with minimum off-target effects through smartly designed carriers. A desirable carrier should be safe, biodegradable, and capable of encapsulating siRNA at the right dose, protecting it from degradation and delivering it to target tissues. Lipid-based nanoparticles (LNPs), such as liposomes, are suitable carriers due to their biocompatibility and ease of preparation.^6,7^ Cationic liposomes have been investigated,^8^ but their instability and toxicity make them a concern for clinical use.^9^ The use of lipid-based particles has been limited by their physicochemical properties.^10^

When used alone, pH-responsive, membrane-active peptides like LAH4 can effectively deliver siRNA into cells by destabilizing endosomal membranes at low pH and this type of peptide has been shown to enhance cell uptake and endosomal escape compared with other systems.^11^ Biophysical measurements of LAH4 and its derivatives show that acidification leads to the release of half the peptides from the DNA cargo, resulting in a self-promoted uptake mechanism where the liberated peptides lyse the endosomal membranes.

A combination of lipids and peptides in LNPs might therefore offer an alternative delivery system with better stability and stronger electrostatic interactions. Indeed, lipid-coated LAH4-L1 particles (LP) have been found effective in delivering nucleic acids to cells.^12^ This was achieved by adsorbing nucleic acids on the surface of the LP particles, which were made of poly(lactic) acid (PLA) coated with a lipid mix, using a layer-by-layer technique. Still, such technologies are yet to be translated to in vivo applications with biodistribution yet to be addressed.

Studies of the biodistribution of siRNA nanoparticles in mice showed that siRNA PEG-polyethylenimine nanoparticles favored liver and kidney distribution.^13^ Combining dynamic positron emission tomography imaging and liquid chromatography–tandem mass spectrometry analysis revealed that 1.62 and 1.70% of the injected dose per gram oligonucleotide was present intact in, respectively, the tumor and liver.^14^ Further, focused ultrasound (FUS) can be used to improve drug targeting by locally heating tissues, increasing particle permeation and drug diffusivity at the sonication site.^15,16^ Packaging siRNA in cationic lipid-polymer hybrid nanoparticles and administering them after the microbubble-enhanced focused ultrasound, results in a more than 10-fold increase of siRNA in the tumor.^17^

**Figure 1:**
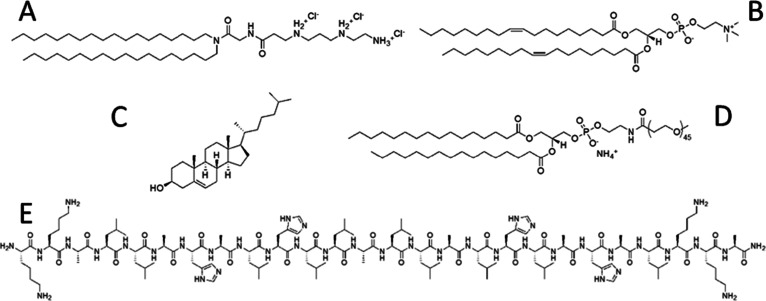
Structures of nanoparticle components. Structures of the lipoplex and ternary complex components: *N′, N′*-dioctadecyl-N-4,8-diaza-10 aminodecanoylglycine amide (DODAG) (A); 1,2-dioleoyl-sn-glycero-3-phosphocholine (DOPC) (B), cholesterol (C); *N*-[methoxy(polyethyleneglycol-2000)]-1,2- distearoyl-sn-glycero-3-phosphoethanolamine (MeO-PEG^2000^-DSPE) (D); and the pH-responsive LAH4-L1 peptide (E).

Therefore, in the present study, we aim to investigate whether the pH-responsive peptide, LAH4-L1, can be combined with PEGylated cationic liposomes for improved endosomal escape and whether the resulting ternary complex is suitable for in vivo application. We used the in-house developed cationic lipid DODAG (*N′, N′*-dioctadecyl-*N*-4,8-diaza-10 aminodecanoylglycine amide; Figure 1) as a key component of the lipid:siRNA and lipid:peptide:siRNA complexes with luciferase and GAPDH used to evaluate specific silencing efficacy in vitro. MDA-MB-231 cell xenograft subcutaneous mice tumor models were used to understand the particle’s pharmacokinetics in real-time using near-infrared fluorescence live imaging. FUS was applied to induce tumor-localized hyperthermia to improve nanoparticle tumor accumulation and uptake. The lipid:Peptide:siRNA complex showed improved gene silencing efficiency in cells, and general labeled siRNA tumor accumulation was observed after FUS in vivo.

## Materials and Methods

### Materials

1,2-Dioleoyl-sn-glycero-3-phosphocholine (DOPC), 1,2-Dioleoyl-sn-Glycero-3-Phosphoethanolamine-N-(Lissamine Rhodamine B Sulfonyl) (DOPE-Rho), and N-[methoxy(polyethyleneglycol-2000)]-1,2-distearoyl-sn-glycero-3-phosphoethanolamine (MeO-PEG2000-DSPE) were purchased from Avanti Polar Lipids (Alabaster, AL). Cholesterol lipid and a custom-made nontargeting siRNA sequence, CUU ACG CUG AGU ACU UCG dTdT, Dulbecco’s modified eagle media (DMEM), OptiMEM, fetal bovine serum (FBS), penicillin and streptomycin, phosphate-buffered saline (PBS), 221 TrypLE express enzyme (1X), bovine serum albumin (BSA), and 4′,6′-diamidino-2-phenyindole, dilactate (DAPI) nuclear staining were obtained from Sigma Aldrich (Poole, UK). N′, N′-dioctadecyl-N-4,8-diaza-10 aminodecanoylglycine amide (DODAG) was synthesized according to the previously published synthetic route.^18^ LAH4-L1 peptide,^11,22^ (Figure 1) amidated at the C-terminus, was purchased from Cambridge Research Biochemicals (Cleveland, UK) as desalted grade (crude). The crude peptide was further purified using water/acetonitrile gradients using a Waters SymmetryPrep C8, 7 μm, 19 × 300 mm column.

1,2-. MTT (3-(4,5-dimethylthiazol-2-yl)-2,5-diphenyltetrazolium bromide), SYBR Safe DNA gel stain, DNA ladder, siRNA loading buffer and E-Gel EX gels, phenol red reagents, GAPDH siRNA (anti-GAPDH and negative siRNA), and KDALERT Ambion GAPDH gene assay kit and Pierce BCA total protein content assay and FAM-labeled Negative Control siRNA were purchased from ThermoFisher Scientific (Oxford, UK). Organic solvents (chloroform, isopropanol, acetonitrile, ethanol, and methanol) and all buffer reagents (HEPES, Tris, sodium acetate, sodium hydroxide) were purchased from VWR (Lutterworth, UK).

A549 cells transfected with episomal plasmid DNA luciferase gene kindly provided by Dr. R. Harbottle.^19^ MDA-MB-231-luc cells were kindly supplied by Prof Takahiro Ochiya (Tokyo, Japan). Luciferase reporter gene assay kit and reporter lysis buffer 5× were obtained from Promega (Southampton, UK). Anti-Luc siRNA-1 for in vitro MDA-MB-231-luc cells studies was obtained from Dharmacon (Cambridge, UK). Silencer Firefly Luciferase (GL2 + GL3) (LucsiRNA) and Silencer Negative Control siRNA #1 for A549-luc cells were purchased from Ambion (Austin, TX). HyLite Fluor-750 labeled siRNA was obtained from Eurogenetec (Camberley, UK).

### Formulation of Lipid:siRNA Complexes

Liposomes were formulated in 4 mM HEPES (pH 7.4) at lipid concentrations of 4 mg/mL. Lipids were stored in chloroform solutions and stored at −20 °C until used. Lipid solutions were only used at room temperature where the needed volume of each lipid was taken and used to mix with other liposomal lipids. Chloroform was then evaporated by a rotary evaporator to create a lipid film. The lipid composition (Figure 1) was DODAG 20%, DOPC 55%, cholesterol 20%, MeO-PEG^2000^-DSPE at 1 or 5% (molar ratio). Following lipid film hydration and five freeze–thaw cycles (freezing by liquid nitrogen and thawing by sonicating at 60 ° C for 5 min), liposomes were extruded through a 100 nm polyethylene membrane using an Avanti manual extruder. The liposomal formulations were then used to encapsulate siRNA at 4:1 positive:negative (+ve:–ve) charge ratio (N:P ratios can be found in Supp Material). The siRNA solution was slowly added to liposomal solutions at a 1:1 v:v ratio and mixed by gentle pipetting. The complexes were incubated at 37 ° C for 15 min before characterization. These lipoplexes comprise the lipid:siRNA formulations at different % of PEG content for either in vitro or in vivo purposes.

### Preparation of Lipid:Peptide:siRNA Complexes

The lipid:peptide:siRNA complexes were prepared at a certain liposome:peptide:siRNA weight ratio. Generally, a range between 1 and 5 μg of siRNA was used. First, the LAH4-L1 peptide was added to the siRNA solution and mixed by gentle pipetting at a 1:1 v:v ratio and incubating at room temperature for 30 min. The mixture was then added to the liposomal solution at a 1:1 v:v ratio. The final mixture was vortexed slightly which was then incubated for 30 min at 37 ° C before biophysical characterization. Weight ratios were varied during the optimization process and the preferred ratio (9:4:1, lipid:peptide:siRNA) was chosen according to acquired size and charge.

### Complexes Size, Zeta Potential, and Morphology

The size of the nanoparticles was characterized by dynamic light scattering at a temperature of 25 °C and at a detection angle of 90° using a Nano ZS apparatus (Malvern Panalytical Ltd., Malvern, UK) after diluting samples with 4 mM HEPES buffer (pH 7.4) to a final concentration of 0.1 mg/mL. For zeta potential (ζ potential), the samples were diluted with 4 mM HEPES buffer (pH 7.4) and ζ potential readings were performed by a Nano ZS apparatus (Malvern Panalytical Ltd., Malvern, UK). Morphological and compositional information regarding the surface of a sample is obtained from AFM. The information obtained from this method was used to indicate the complexes’ diameter and its shape in its normal physical state. Prior to imaging, 1 mg/mL solution of the siRNA complexes was prepared, and approximately 10 μL was pipetted onto a fresh mica sheet (1 cm × 1 cm) and dried using nitrogen gas. Images were taken using the Bruker Dimension Icon atomic force microscope, with the standard tapping mode applied. The scanning area started at a large region of 10 × 10 μm and decreased to a smaller region of interest (ROI) of 1.66 × 1.66 μm. The scan rate decreased from 0.99 to 0.70 Hz as the scan size decreased, to improve image quality. The software uses a proportional–integral–derivative (PID) feedback system to improve the trace and retrace signals. To improve the reliability of image acquisition, the scan angle of the probe is altered from 0° to 90° and the raster scan for the images is repeated. This is a confidence check to observe any artifacts produced because of particles/dust stuck onto the cantilever. Images were then processed using Gwiddione software for visualization.

### Encapsulation Efficiency

Encapsulation efficiency was tested by PicoGreen assays in black 96-well plates. For each well, 50 μL of PicoGreen reagent [1:199 v:v diluted with Tris-EDTA (TE) buffer, pH 7.4] was used. For the free siRNA fluorescent intensity, 3 wells were loaded with 50 μL of 3 μM of naked negative control siRNA in TE buffer (giving a final siRNA content of 150 pmol per well). The (complexes samples) wells were loaded with 50 μL of liposomes or ternary complexes which were prepared by complexing 300 pmol siRNA per 100 μL sample (150 pmol per well). Fluorescence was measured after 10 min incubation at room temperature and at λ_ex_/λ_em_ = 480/520 nm using an Infinite 200 PRO spectrofluorimeter (TECAN trading AG, Mannedorf, Switzerland). In each experiment, negative control siRNA mixed with PicoGreen was used as 100% fluorescence intensity signal and was used to normalize the PicoGreen signal detected from the complexes. The background measurements were considered as the fluorescence intensities of PicoGreen solution. Encapsulation efficiency was measured as percentage and calculated by the following formula: % siRNA encapsulation = (1 – ([*F*_(complex)_ – *F*_(PG)_]/[*F*_free siRNA_) – *F*_(PG)_])) × 100, where *F*_complex_ = fluorescence intensity of siRNA intercalated with PicoGreen after encapsulation, *F*_free siRNA_ = fluorescence intensity of free siRNA intercalated with PicoGreen (nonencapsulated), and *F*_(PG)_ = background reading of PicoGreen solution.

### Gel Retardation

Gel electrophoresis was used to visualize (1) the ability of complexes to bind siRNA, (2) test the release of siRNA, and (3) assess the effect of different serum conditions on the stability of complexes. siRNA was complexed with the samples at a final volume of 20 μL (20 pmol siRNA per sample). Samples were then vortexed slightly and incubated at 37 °C for 15 min. For encapsulation and release studies, complexes were loaded into 4% agarose gel. Electrophoresis was performed using Invitrogen E-Gel EX 226 pre-cast Agarose Gels Electrophoresis System (Invitrogen Corporation, Carlsbad, USA) for 30 min and the intact siRNA was visualized by a fluorescent nucleic acid stain (λ_ex_/λ_em_ = 490/520) incorporated into the gels. All samples contained 20 pmoles of siRNA in a final volume of 20 μL. Six different media conditions for each sample were tested: full H_2_O, full OptiMEM, full serum (FCS) containing media (MEM, 10% FBS), water/OptiMEM (50%:50%), water-/serum-containing media (50% water + 50% MEM) and water/10% FBS. For the last three, samples were prepared initially in RNAse-free H_2_O (in the 12.6% v/v of the final volume for lipid:siRNA and peptide:siRNA complexes, and 21% v/v for lipid:peptide:siRNA complexes) and then diluted in OptiMEM, serum-containing growth media (MEM) or 10% fetal bovine serum (FBS) in water. Free siRNA prepared under those six conditions was used as a control. After incubation at 37 ° C for 30 min, samples were mixed with 1 μL of GelRed Nucleic Acid Gel Stain diluted 10,000-fold in water (Biotium, CA) and loaded into a 2% agarose gel. The gel was run for 40 min at 80 V using a Sub-Cell GT Agarose Gel Electrophoresis Systems (Bio-Rad laboratories, Watford, UK) and visualized under UV light.

### Stability of siRNA Nanoparticles in High Content-Serum: Turbidity Studies

siRNA complexes were prepared as previously stated using 300 pmol siRNA. After preparing the mixtures, nanoparticles were diluted to 500 μL in FBS and incubated in flat-bottomed 24-well plates at 37 °C for up to 12 h. Absorbance at λ = 600 nm was measured every 10 min using the Infinite 200 PRO fluorimeter (TECAN trading AG, Mannedorf, Switzerland).

### FACS Studies on siRNA Complexes Uptake

Using the 1 mol % PEG, 5 mol % PEG lipid:siRNA, LAH4-L1:siRNA complex, and lipid:peptide:siRNA ternary complexes described earlier, FAM-siRNA was added at 4:1 charge ratio, under vortex, to form siRNA nanocomplexes. After encapsulation of FAM-siRNA, nanoparticles were suspended in OptiMEM. MDA-MB231 cells were seeded in 12-well tissue culture plates at 4 × 10^4^ cells/well and grown until 80% confluency. A549 cells were seeded in 12-well tissue culture plates at 5 × 10^4^ and grown until 80% confluency. The growth media was removed and replaced by nanocomplexes in OptiMEM at 50 nM concentration. After incubations of 4 h, nanoparticles solutions were removed and cells were washed with PBS. Cells were dislodged by trypsin and collected by centrifugation. Trypsin-containing supernatant was removed, and the cell pellet was suspended in PBS at 1 × 10^6^ cells/mL, 2% of FBS was included to maintain cell viability and to avoid cell clumping. Cell suspensions were analyzed by flow cytometry for fluorescence in the FL-1 channel using the Cytomics Fc500 Flow Cytometer (Beckman Coulter Inc., Brea, CA). Histograms were analyzed using FlowJo software (FlowJo LLC, Ashland, OR).

### Confocal Imaging Microscopy

A549-luc and MDA-MB231 cells were seeded 2 × 10^4^ cells per well on 8-well (Falcon Chambered Cell Culture Slides, Fisher Scientific, UK) or 16-well plates and grown at 37 °C in a 5% CO_2_ and 90% relative humidity, until 80% confluency was reached. For labeling, FAM-labeled siRNA or Rhodamine B was alternatively used for each control. All samples were initially prepared in Nuclease-free water (10% v/v of final volume) and diluted with OptiMEM to have a final siRNA concentration of 70 nM per well. After incubation at 37 °C for 4 h, samples were washed twice with DPBS. DAPI nuclear staining solution (300 nM) was added to each well. After 15 min of incubation at 37 °C, cells were washed 3 times with PBS. Cells were then fixed with 3% paraformaldehyde solution (incubation for 15 min at 37 °C). Cells were then washed and mounted using DPX Mountant (SigmaAldrich Company Ltd., UK) on Fisherbrand borosilicate glass square coverslips and left overnight to dry at 4 °C. Samples were imaged by confocal microscopy using the Nikon A1 inverted confocal microscope (Nikon Instruments Inc., Melville, NY).

### Cell Viability

A549-luc and MDA-MB231-luc cells were seeded in the 96-well plate at 12 × 10^3^ cells per well (A549-luc) or 10 × 10^3^ cells per well (MDA-MB231-luc) and grown until they reached 70% confluency. Growth media was removed and replaced by complexes containing OptiMEM. After 4 h of incubation cells were washed again and new growth media was added for 48 h. 10 μL of 12 mM MTT (3-(4,5-dimethylthiazol-2-yl)-2,5-diphenyltetrazolium bromide) stock solution were added to each well and incubated for 4 h at 37 °C. Cells were then washed and treated with DMSO for 1 h. Absorbance was measured at 570 nm using an Infinite 200 PRO Spectrofluorometer (TECAN Group Ltd., Mannedorf, Switzerland). Lipofectamine RNAiMAX (ThermoFisher Scientific, Basingstoke, UK) and untreated cells were used as controls.

### In Vitro siRNA Delivery and Silencing Studies

Luciferase and GAPDH were silenced in both A549-luc and MDA-MB-231-luc cells using anti-luciferase siRNA and GAPDH siRNA. A549-luc and MDA-MB231-luc cells were seeded, respectively, at 12 × 10^3^ and 10 × 10^3^ cells/well and grown for 48 h to reach a confluency of 70%. 20 μM Silencer Firefly Luciferase (GL2 + GL3) stock solution was used in A549 luciferase knockdown experiments. Anti-luc siRNA 1 was used in MDA-MB-231-luc luciferase knockdown experiments. Ambion Anti-GAPDH siRNA was used for both cell lines in the GAPDH knockdown experiments. Lipofectamine RNAiMAX (ThermoFisher Scientific, UK) was used as control. Silencer-negative siRNA was used as a negative control for each condition. Samples were prepared following the usual method in RNAase-free H_2_O (Nuclease-free Water, ThermoFisher Scientific, Basingstoke, UK) in 10% of the final volume and then diluted in OptiMEM. A549-luc cells were transfected with siRNA-encapsulating complexes at the final siRNA concentration of 70 nM. MDA-MB-231-luc cells were transfected by complexes encapsulating siRNA at a final concentration of 70 nM. Cells were washed after 4 h of transfection and 100 μL of fresh growth media were added. In the case of the luciferase silencing studies, cells were washed twice after 48 h of transfection using DPBS and lysed using passive lysis buffer provided by the luciferase assay kit (Promega, UK). Luciferase expression assay was performed using a manual luminometer (Lumat LB 9507, Berthold Technologies) following instructions provided by the luciferase assay kit. In the case of the GAPDH silencing, 48 h after the transfection, cells were lysed with KD alert lysis buffer and cell lysis solutions were added to assay solutions and fluorescence at λ = 560/590 nm ex/em was measured at *t* = 0 min and *t* = 4 min after mixing with assay solutions. GAPDH protein content was calculated according to correlation with the linear GAPDH calibration curve. %GAPDH knockdown = FluorGAPDH siRNA and Fluor-negative siRNA corresponds to the fluorescence readings of post-assay cell lysates of cells treated with anti-GAPDH siRNA and negative siRNA, respectively.

### In Vivo Murine Tumor Model

All animal experiments were approved by the King’s College London ethical review committee and conducted under Home Office Project License P851CE254. MDA-MB-231-luc cells (5 × 10^6^) were suspended in 100 μL PBS/Geltrex Reduced Growth Factor Basement Membrane Matrix (50:50 v:v). Cells were then inoculated subcutaneously into SHO (SCID hairless outbred) mice which were used for FUS experiments to avoid the formation of air bubbles and the reflection of ultrasound. No difference in the biodistribution behavior between both strains was observed. Mice were anesthetized with isoflurane and tumor growth was monitored every 24 h for 10 to 14 days until stable tumors were formed. Experiments were carried out when tumor diameter reached 5–6 mm.

### In Vivo Biodistribution Studies

For the lipid:siRNA biodistribution experiments, liposomes 5 mol % PEG were prepared in 20% glucose/10 mM HEPES buffer pH 7.4 at 18 mg/mL concentration and mixed with siRNA (in water) at 1:1 v:v ratio to formulate lipoplexes at a final concentration of 9 mg/mL in 10% glucose 5 mM HEPES buffer. HiLyte Fluor-750-labeled siRNA (100 mM) was encapsulated to give a final dose of 2.5 mg/kg (200 nmol/kg). In the case of lipid:peptide:siRNA biodistribution studies, liposomes 5 mol % PEG were prepared in 20% Glucose/10 mM HEPES buffer pH 7.4 at 16 mg/mL concentration (*n* = 3 mice). HyLite Fluor-750-labeled siRNA suspended in RNAse-free water (100 mM) was complexed with LAH4-L1 peptide in RNAse-free water (10 mg/mL) and mixed with liposomes at 1:1 v:v ratio. A final dose of 1.25 mg/kg (100 nmol/kg) siRNA was used (*n* = 2 mice). An average weight of the mouse was 25 g. Mice were anesthetized with isoflurane and formulation was administered intravenously through tail vein. Biodistribution was followed for 24 h by NIR imaging of the HyLite Fluor-750 dye by a MAESTRO-EX (Perkin Elmer, MA, USA) in vivo imaging system. The NIRF signal was scanned at a wavelength range (720–900 nm) for 500 μs exposure time. The MaestroEX settings were adjusted to record the Hylite (750 nm) fluorescence signal. Finally, the images were unmixed (multispectral analysis) using the Maestro 3.00 software, and balanced using ImageJ.^20^ Signal intensity or NIRF brightness from regions of interest (e.g., center of the tumor) of left and/or right tumors were presented. The organ fluorescence was imaged from excised organs at 24 h postinjection.^21^

### Effect of Focused Ultrasound on Tumor Biodistribution of Lipoplexes and/or Ternary Complexes of FUS

One tumor (right side unless otherwise stated) was treated with FUS-induced hyperthermia using a Therapy and Imaging Probe System (TIPS) (Philips Research, Briarcliff NY, USA), The second tumor was not exposed to FUS and was used as a control. Treatment was applied under isoflurane anesthesia and mice were located on a warmed gel pad over an ultrasound-absorbing mat. Two fine-wire thermocouples (T-type, 40 ga, Physitemp Instruments Inc., Cifton NJ, USA) were implanted on the top and below the targeted tumor and temperatures recorded (0.1 °C, 0.1 s resolutions) during the treatment. Ultrasound gel was used to cover the tumor. TIPS was placed at 80 mm from the skin surface of the tumor. FUS insonation was carried at 1.0 MHz frequency and 99.9% cycle duty, and 12–15 W of acoustic power was actively adjusted to reach a temperature of 42 °C. Insonation was maintained for 3 min without further temperature increase.

## Results and Discussion

### Complexes’ Size and Polydispersity Is Reduced by Increasing the Proportion of LAH4-L1 Peptide and DODAG Relative to siRNA

**Figure 2:**
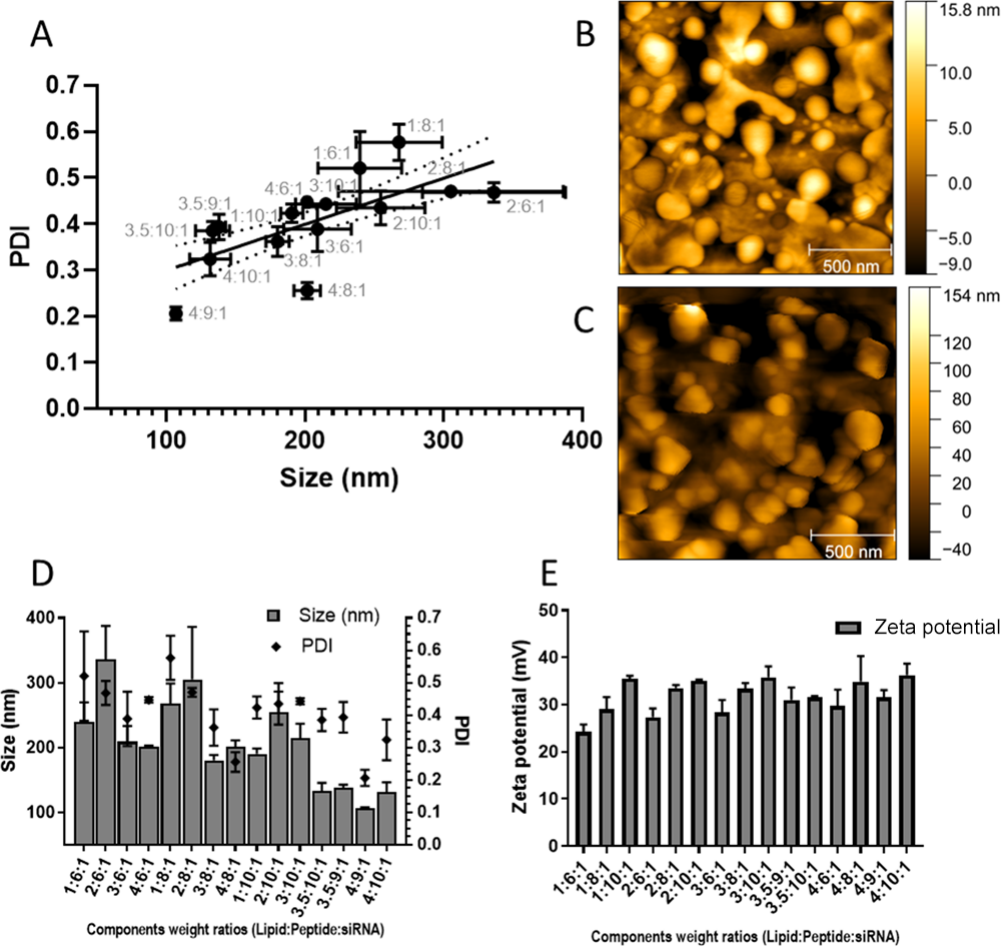
Physicochemical characteristics of the lipid:peptide:siRNA complexes. Increasing the lipid and peptide to siRNA weight-to-weight ratio leads to decreased complex size and polydispersity index (PDI) (D) and these two measures are correlated (A; Spearman *R* = 0.800; *p* = −0.0006). The zeta potential of the complexes (E) was largely unaffected by the formulation and this parameter did not correlate with size or PDI (*n* = 3; ±SD). AFM images of peptide:siRNA (B) and lipid:peptide:siRNA (C) show agreement with the size range obtained from DLS. A table giving the N:P ratios for the ternary complexes is provided in the Supplementary Material.

**Figure 3:**
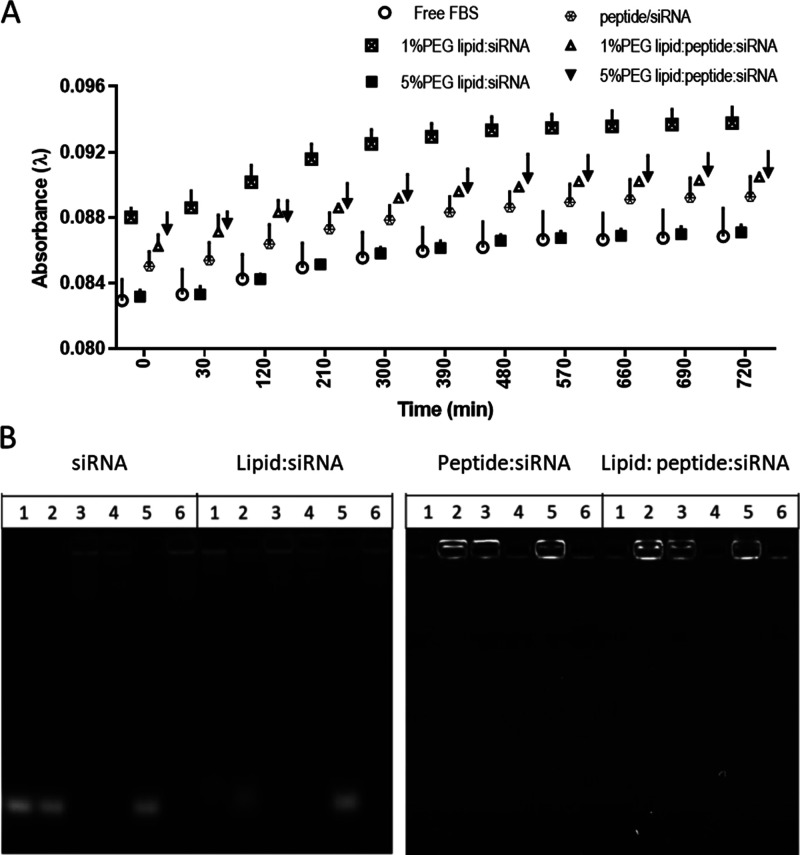
Stability assessment of Pegylated lipid:peptide and lipid:peptide:siRNA complexes. (A) Aggregation of complexes in 50% v/v serum in the buffer. Increased absorbance (turbidity) indicates if nanoparticles aggregate over time. (*n* = 3; ± SD) (B) gel electrophoresis images of lipid:siRNA, peptide:siRNA complex, lipid:peptide:siRNA complex incubated with different media to test the complex stability. (1) Prepared fully in water, (2) prepared in 10%v/v FBS aqueous solution, (3) prepared in OptiMEM, (4) prepared in 10%v/v FBS containing medium (MEM), (5) fully complexed in OptiMEM, and (6) fully complexed in serum-containing medium (MEM).

**Figure 4:**
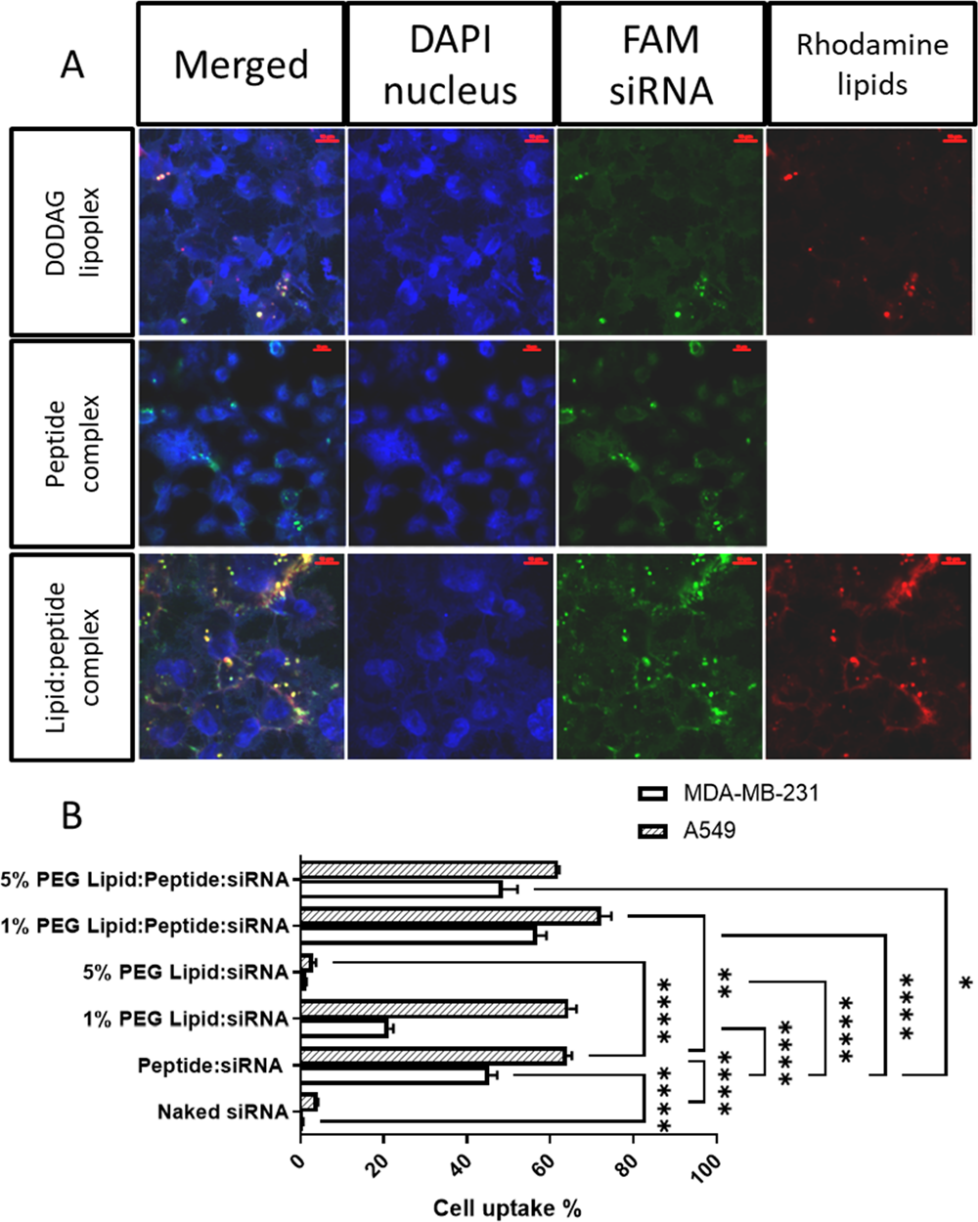
LAH4-L1 promotes siRNA complex uptake in vitro even in the presence of PEGylated lipids. (A) Maximum projection of confocal fluorescence microscopy cross sections of MDA-MB231 cells treated with 70 nM FAM-siRNA (green) complexes containing Rhodamine-lipid (red) while cell nuclei are stained with DAPI (blue). The scale corresponds to 20 μm. (B) Flow cytometry analysis corresponding to the uptake of FAM-siRNA complexes (peptide:siRNA 10:1 wt:wt; lipid:peptide:siRNA 4:9:1 wt:wt:wt) (*n* = 3; ± SD). Statistical analysis is by Two-way ANOVA with multiple comparisons correction by Benjamini, Krieger, and Yekutieli false discovery rate control. All comparisons relative to peptide:siRNA are shown where *p* < 0.05; *p < 0.05; **p < 0.01, ***p < 0.001; ****p < 0.0001.

LAH4-L1 is a pH-responsive peptide that can complex with either DNA or siRNA and is partially released from such complexes during endosomal acidification where it actively disorders and hence disrupts the endosomal membrane.^22,23^ While it is itself an effective mediator of in vitro plasmid DNA or siRNA transfection, it has not yet been formulated together with cationic lipids. Here, we investigate its effect on cationic liposomal siRNA complex formation (Figures 2 and 3) and subsequent in vitro transfection properties (Figures 4^5^,6).

**Figure 5:**
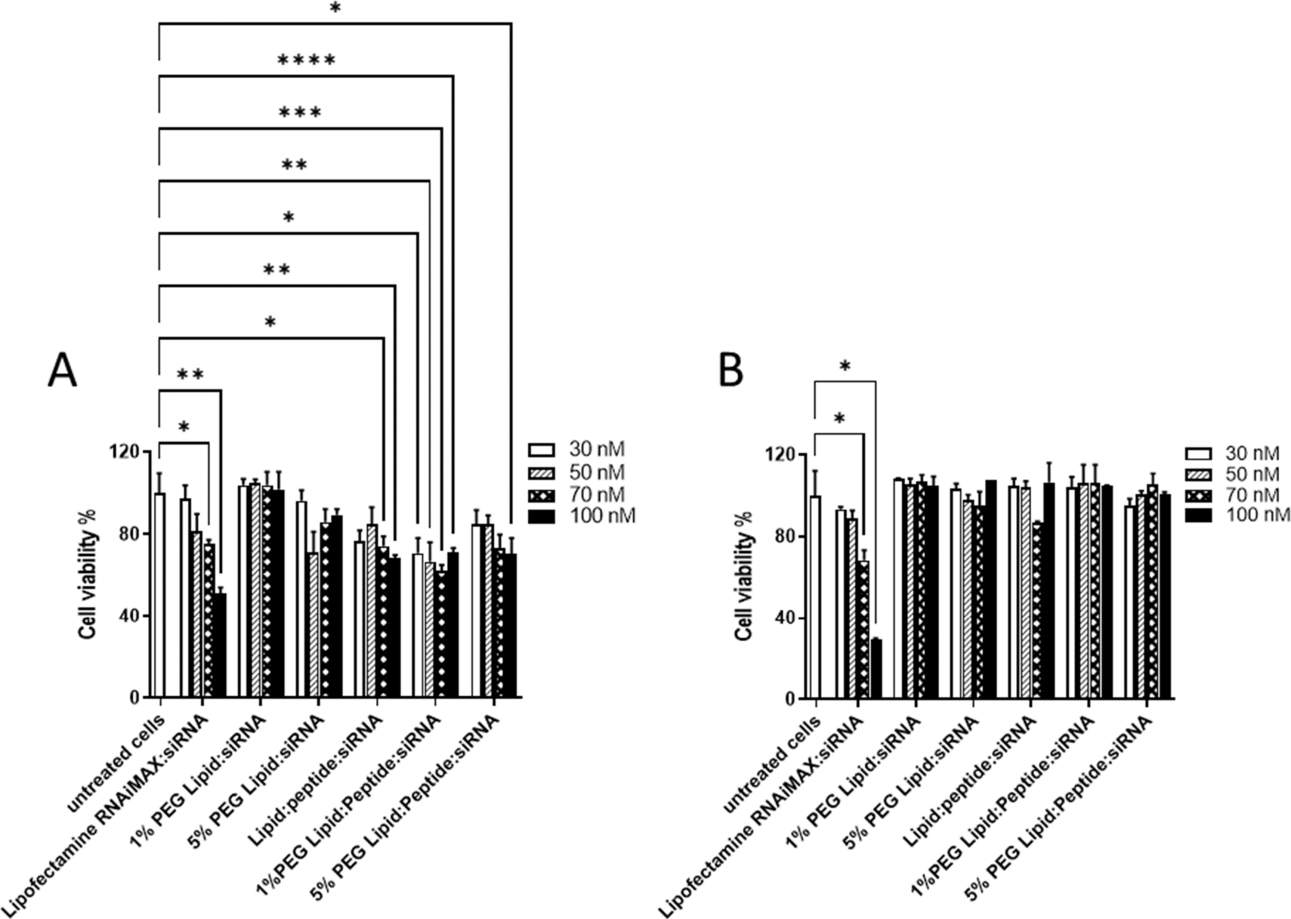
Cell viability after incubation of siRNA complexes and RNAiMAX siRNA complexes. Cells were analyzed at 48 h posttransfection of MDA-MB-231 (A) A549 (B) cells. (*n* = 3 ± SD).

**Figure 6:**
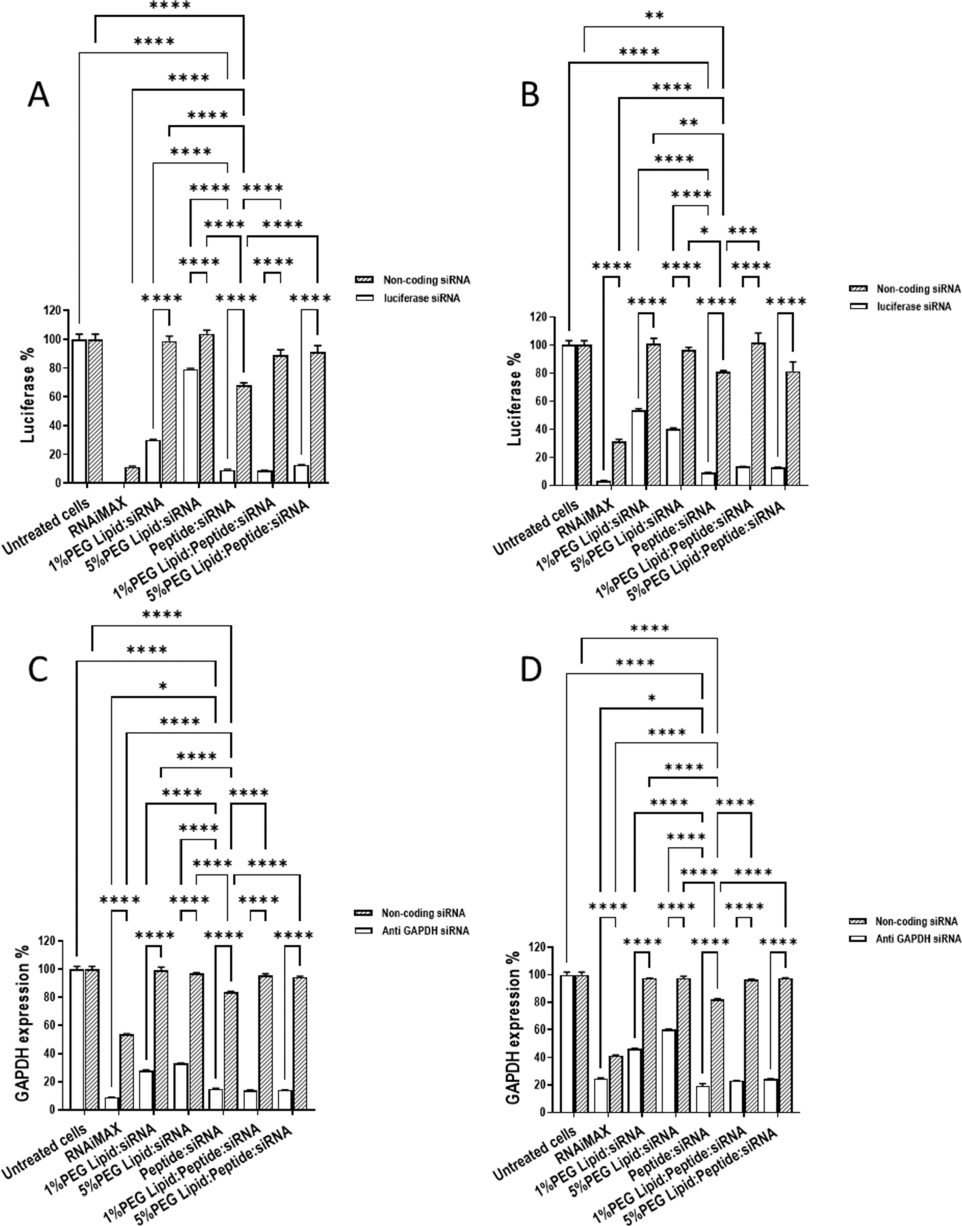
Ternary complexes mediate highly effective and specific gene silencing. siRNA transfection efficiency tested on MDA-MB-231 (A, C) and A549 cells (B, D) by silencing luciferase (A, B) or GAPDH (C, D). siRNA (negative control) was at 70 nM siRNA. Cells were co-incubated with siRNA at 70 nM (*n* = 3 ± SD).

Cationic liposomes were prepared using the DODAG lipid as previously described^18^ to allow comparison of lipid:siRNA and lipid:peptide:siRNA complexes. LAH4-L1 was used with the aim of improving the endosomal escape of the complex and the overall silencing effect.^24^ LAH4-L1 peptide/siRNA complexes are used as a reference in most in vitro studies. Lipid:siRNA complexes were prepared at 10 mg/mL lipid concentration encapsulating siRNA at 4:1 +ve:–ve charge ratio. The resulting lipoplexes had an average particle size of 83.7 ± 5 nm, PDI of 0.197 ± 0.01, and zeta potential of 15.4 ± 3.4 mV. LAH4-L1 was complexed with siRNA at a 10:1 peptide:siRNA weight:weight ratio resulting in complexes of average particle size 122.2 ± 1.3 nm and zeta potential +24.2 ± 2.0 mV.

For the lipid:peptide:siRNA complex, 15 different weight ratios were prepared by adding the peptide slowly to the siRNA solution at a 1:1 v/v ratio. Cationic liposomes were added to the mixture at a 1:1 v:v ratio, and the resulting complexes were characterized (Figure 2). At higher lipid:peptide to siRNA ratios, the particle size and polydispersity decreased (Figure 2B) and these two parameters were strongly correlated (Figure 2A). AFM images were obtained in support of the DLS and the corresponding data are consistent with the size range and polydispersity (Figure 2B/C). At a 4:9:1 weight ratio, the lipid:peptide:siRNA ternary complex forms small particles with an average particle size of 115 ± 10 nm (Figure 2D) and a zeta potential of +29.0 ± 2.3 mV (Figure 2E). The zeta potential did not vary substantially according to the varying DODAG or LAH4-L1 content (Figure 2E).

### LAH4-L1 Increases Complex Stability

The siRNA encapsulation efficiency of the various complexes was tested by gel electrophoresis and PicoGreen fluorometric assays (Figure S1A). A high level of encapsulation was observed by both gel electrophoresis and PicoGreen assays where it was found to be more than 90%. The encapsulation efficiency was maintained under serum conditions. Then, the potential of recovering siRNA from the liposomal particles was assessed by solubilizing the lipidic elements of the particles using Triton X-100. This method aimed to identify the integrity of siRNA during formulation conditions. No release of siRNA from the particles occurred with the use of the surfactant at 1% (v/v) final concentration until heat was applied at 37 °C. After mixing the lipoplexes with 2% Triton X-100 solution at 1:1 v:v ratio and incubating the mixture at 37 °C for 15 min, samples were checked by Gel electrophoresis 4% agarose gel. The recovery of siRNA from lipoplex showed a clear complete release (Figure S1B). However, when the same method was applied for peptide-siRNA complexes and lipid:peptide:siRNA complexes, no release was seen (Figure S1B).

### Pegylated siRNA Complex Stabilities in Serum

Buffers with serum have been used as a model that mimics blood and are used here to investigate the ability of siRNA complexes to avoid aggregation and the effect of incorporating PEGylated lipids. With a diameter of around 100 nm, aggregation of nanoparticles will cause a reduction in light transmission at λ = 600 nm due to increased light scattering by the colloids. The effect of serum on the aggregation of the complexes was monitored for 12 h with previous studies indicating that cationic siRNA nanoparticles aggregate in serum media even at 2%v/v serum.^25^ Here, we observe a gradual increase in absorbance at 600 nm irrespective of the presence of siRNA-containing nanoparticles. For DODAG/siRNA liposomes, the effect of PEG mol % on aggregation is striking as 1% PEG lipid:siRNA containing complexes have a substantially higher absorbance, while that for 5% PEG lipid:siRNA complexes is equivalent to that of the serum-only control (Figure 3A). This is consistent with a previously observed phenomenon^26^ where increasing the mol % DSPE-PEG improves the colloidal stability of complexes in serum. However, such a difference is not observed for lipid:peptide:siRNA complexes where increasing the mol % of PEGylated lipids has no effect on the absorbance (Figure 3A). Notably, however, the absorbances of LAH4-L1 containing ternary complexes are intermediate to those obtained for the 1% PEG and 5% PEG lipoplexes (Figure 3A).

### LAH4-L1 Improves Complexation and siRNA Protection in Pegylated siRNA Complexes

Gel electrophoresis studies were performed to study complexation and stability of the nanoparticles under various conditions (Figure 3B). The detected bands at the bottom of the gels represent the free intact siRNA. Naked siRNA shows no band when it is placed under serum-containing conditions reflecting the degradation of the siRNA by serum RNAse (wells 3, 4, 6; Figure 3B). siRNA stays intact when mixed in water before the addition to serum-containing media (contains 10% FBS) or (FBS-free) OptiMEM as clear bands are observed for these conditions at the bottom of the gel (wells 1, 2, 5; Figure 3B). Slight degradation of the siRNA was detected when the preparation was made directly in serum-containing media as a very vague streak was observed on the gel (wells 3, 4, 6; Figure 3B). Although lipid:siRNA complexes are shown to complex siRNA efficiently in water, as a very light band was observed at the bottom of the gel for that condition, some siRNA release (and possible degradation) is observed under other conditions tested. This can be observed as partial or full leakage of the siRNA in wells 2 and 5 and streaks along the gel for wells 3, 4, and 6. The exact same pattern appears in the case of the free siRNA and the lipid:siRNA samples, reflecting a minimal protection of the siRNA from serum conditions when encapsulated with liposomes at body temperature for 30 min. Interestingly, LAH4-L1 peptide:siRNA complexes and lipid:peptide:siRNA complexes complex and protect siRNA to a higher extent than that observed for lipid:siRNA, as the siRNA band did not move along the gel. When comparing the complexation achieved and considering the colloidal stability, it can be concluded that the ternary lipid:peptide:siRNA complexes demonstrate the highest degree of siRNA complexation under serum-containing conditions.

### LAH4-L1 Enhances Complex Uptake and Functional siRNA Delivery

Confocal microscopy (Figure 4A) and flow cytometry (Figure 4B) studies of uptake of fluorescein amidite (FAM)-labeled siRNA in lipid:siRNA, peptide:siRNA, or lipid:peptide:siRNA (4:9:1 wt:wt:wt) complexes were performed on MDA-MB-231 and A549 cell lines. Intracellular localization of siRNA is observed in cells transfected with each of the three different complexes though the fluorescent-labeled siRNA signal observed in cells transfected by lipoplex is low (Figure 4A).

Co-localization between siRNA and lipids (seen in yellow; Figure 4A) is observed with cells transfected by lipoplex and the lipid:peptide:siRNA complexes. This co-localization indicates the presence of siRNA in the cytosol (around the nuclei shown in blue, also see Supp Material Figure S3). Nonencapsulated siRNA-treated cells do not show cell-associated fluorescence. Only the siRNA that was encapsulated within complexes exhibits cell-associated fluorescence, due to the cationic, fusogenic, and/or lipidic nature of the nanoparticles, which assists cell-binding and endocytosis. The peptide:siRNA complexes show high uptake percentages (Figure 4B), likely attributable to the LAH4-L1 peptide, which could be attributed to interactions with the cellular membrane.^27^ PEG contributes to colloidal stability and helps the complexes circulate in the blood,^28^ but it can also cause diminished nanoparticle endocytosis.^29^ Therefore, as expected, the addition of PEG to lipid:siRNA complexes reduces cell uptake, while uptake of 1% PEG lipid:siRNA complexes is comparable to peptide:siRNA in A549 cells and only a little lower in MDA-MB-231 cells (albeit significantly so; *p* < 0.0001), 5% PEG complexes show practically no uptake (Figure 4B). In contrast, ternary lipid:peptide:siRNA complexes display excellent cellular uptake even when coated with 5 mol % PEG. While there is better uptake of 1% PEG lipid:peptide:siRNA complexes than either peptide:siRNA or the corresponding lipid:siRNA complexes in both cells (*p* < 0.002), the difference is most stark for the 5% PEG complexes where lipid:peptide:siRNA complexes clearly outperform their lipid:siRNA counterparts and are either noninferior to (A549 *p* = 0.2101) or slightly better than (MDA-MB-231 *p* = 0.0394), peptide:siRNA complexes (Figure 4B).

### Ternary Complexes Are Well Tolerated by Cells

siRNA complexes were assessed for their toxicity profiles on cells using MTT assays on both A549 and MDA-MB231 cells using a range of siRNA concentrations (30, 50, 70, and 100 nM). Lipofectamine RNAiMAX, a commonly used transfection agent, was included for comparison. The MTT assays show a significant, dose-dependent, cytotoxic effect for RNAiMAX relative to untreated control cells (Figure 5). Generally, all the complexes were found to be less toxic than Lipofectamine RNAiMAX. However, lipid:siRNA complexes at 1% PEGylation were shown to be the least toxic as they did not significantly affect cellular viability.

This may be due to the low cationic charge compared to the other complexes. A slight decrease in the cell viability was observed with the lipid:peptide:siRNA complexes. However, the cell viability of MDA-MB231 cells was observed to be affected more than A549. Studying the cell viability using two different cell lines provided confidence in the findings. As anticipated, each formulation affected cell viability in a different manner (Figure 5). MDA-MB231 cells seemed to be more affected with reduced viability compared to A549 for all treatments.

The ability of different formulations to induce siRNA knockdown was examined (Figure 6) and is considered with respect to the relative uptake (Figure 4B) of, and toxicity (Figure 5) due to, the different complexes. Specific silencing of luciferase (Figure 6A/B) or GAPDH (Figure 6C/D) is observed under all conditions bar one—expression of luciferase in MDA-MB-231 cells is so low when noncoding RNA was delivered with RNAiMAX, likely due to toxicity, that there is no significant difference when compared with luciferase expression in the corresponding transfection where antiluciferase siRNA was delivered (Figure 6A). In the remaining studies, although very substantial nonspecific silencing is also observed when noncoding siRNA is delivered by RNAiMAX, the impact of the antiluciferase or anti-GAPDH siRNA is significant (Figure 6B–D). In contrast, although LAH4-L1 also mediates nonspecific silencing under the same conditions, this is less severe, and the specific silencing is very considerable (Figure 6). LAH4-L1:siRNA achieves a very high silencing effect (≥90% luciferase knockdown and ≥85% GAPDH knockdown) in both cell lines.

Neither lipid:siRNA nor lipid:peptide:siRNA complexes induce toxicity in A549 cells when incorporating either 1 or 5% PEG (Figure 5B) and they are also well tolerated by MDA-MB-231 cells (Figure 5A). Variation in gene silencing ability by these complexes may therefore be expected to be related to uptake efficiency. Indeed, the specific silencing mediated by either 1 or 5% PEG lipid:siRNA complexes is consistently lower than that effected by the corresponding ternary complexes (Figure 6A–D) which had better uptake (Figure 4B). However, closer inspection of the data suggests the silencing efficacy of these systems is only partly dependent on their cell uptake. Notably, while 5% PEG seems to almost abolish completely the lipid:siRNA complex uptake, some modest specific gene silencing is observed which, with the exception of luciferase knockdown in MDA-MB-231 cells, is similar to that achieved with the 1% PEG lipid:siRNA complexes which are taken up relatively well. On the other hand, uptake of 5% PEG lipid:peptide:siRNA is no greater than that observed with 1% PEG lipid:siRNA in A549 cells (Figure 4B) but the silencing of both luciferase and GAPDH expression is much more effective (Figure 6B/D). Taken together, this suggests that silencing can be a very inefficient process, with substantial uptake not necessarily translating to effective silencing, and that combining LAH4-L1 with DODAG and siRNA increases silencing efficiency. Future work may involve examining dose-dependent uptake to ascertain whether the observed differences in performance of the various complexes can be exacerbated and the advantages of the ternary complexes made even clearer.

In summary, the in vitro transfection studies show that combining LAH4-L1 peptide with DODAG liposomes offers a safe and efficient siRNA delivery system even when the complexes are coated with 5 mol % PEG. The formed complexes are of about 100 nm size with a 30 mV positive zeta potential, lack significant cytotoxicity, and yet achieve substantial gene silencing in vitro in cells. This improvement over lipid:siRNA complexes is likely due to both improved cellular uptake and the fusogenic/endosomolytic properties of the peptide which have been previously studied.^27^ As a result, luciferase knockdown achieved by the lipid:peptide:siRNA complex is comparable with that reported previously by Kudsiova et al., where similar cationic lipid and cationic peptide formulations were used in A549 cells,^30^ while both luciferase and GAPDH knockdown is comparable with that reported by Tagalakis et al. using similar liposomal/peptide complexes.^31,32^

### Near Infrared Fluorescence Imaging Reveals Strong Accumulation of siRNA Complexes in Tumor Xenografts Following Application of Focused Ultrasound

Although the lipid:peptide:siRNA formulations demonstrate safety and strong efficiency in vitro, it remains a question as to whether these complexes can be taken up by tumors in animal models. RNA is inherently unstable and potentially immunogenic and hence requires a delivery vehicle for efficient transport to the targeted cells. These issues have hindered the clinical progress of some RNA-based drugs and have contributed to mixed results in the clinic.^33^ Cationic vesicles stabilize siRNA, but most cationic nanoparticles are subject to rapid clearance by the reticuloendothelial system and poor tumor permeation.^34^

For decades, it has been observed that hyperthermia has a synergistic effect with neoadjuvant chemotherapeutics and radiotherapy in the clinic, improving the treatments.^35,36^ Tumor vascular permeability presents one of the important challenges which needs to be overcome to improve drug delivery. Local hyperthermia increases the pore size in tumor vasculature, reduce hydrodynamic burdens, like those stimulated by the intratumoral interstitial fluid flow and pressure which could lead to nanoparticle (∼100 to 150 nm in diameter) extravasation.^37^ Hyperthermia can also increase local blood perfusion which could help modify the pharmacokinetics of any delivery system in the heated area. Such effects of hyperthermia were previously described.^38,39^

**Figure 7:**
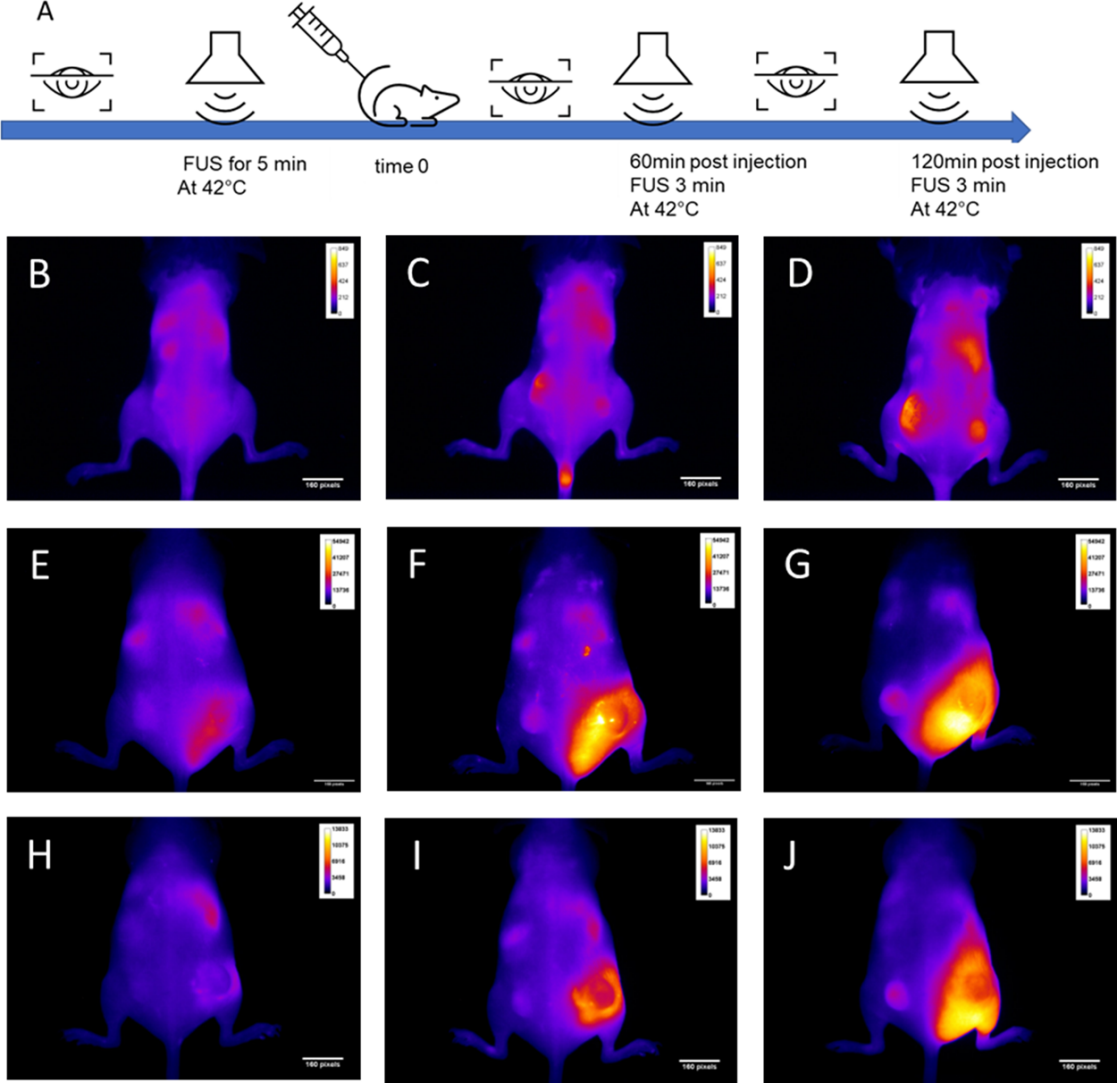
Focused ultrasound enhances tumor uptake of complexes carrying near-infrared fluorescently labeled siRNA. SHO mice bearing two tumors at either flank were administered 5% PEG lipid:siRNA (B–G) or 5% PEG lipid:peptide:siRNA (H–J) carrying Hylite-labeled siRNA (1.25 mg/kg siRNA). (A) scheme of time and sequence of events (i) injection (ii) FUS increases tumor temperature to 42 °C. When FUS is applied to the right flank tumor (E–J), there is a substantial change in biodistribution compared to i.v. administration in the absence of FUS (B–D). Images recorded at 45 min (B, E, H), 180 min (C, F, I), or 24 h (D, G, J) post i.v. administration. Images of excised organs are available in Figure S4.

Here, we present a preliminary investigation of whether the issue of poor tumor uptake might be addressed by using focused ultrasound to increase the tumor temperature up to 42 °C for a brief period (Figure 7A) with timings of the application based on previous experiments.^21^ SHO mice with two tumors at either flank were injected intravenously with lipid:siRNA or lipid:peptide:siRNA with the siRNA labeled with Hylite near-IR fluorescent tag and imaged in real time and at regular intervals. In this experiment, mice developed similar size tumors (left right and between animals) and were administered the complexes via an intravenous administration. Therefore, uptake by either tumor (left/right) is theoretically the same if no FUS enhancement is applied, while this is a controlled experiment that avoids the potential different particle kinetics (inter mice variability). Whole animal imaging at three time points postinjection and treatment is presented alongside images of key, excised organs obtained from animals sacrificed 24 h postinjection (Figures 7 and S4). At 180 min of the intravenous administration of the lipoplexes, and shortly after the last ultrasound treatment, the complexes distribute mainly in the tumor that was treated by FUS, while the untreated tumor of the animals (left flank) show no siRNA-related NIRF signal. Therefore, although the preliminary study was performed in a limited number of animals, imaging clearly shows that focused ultrasound treatment leads to the substantial accumulation of the siRNA complexes in treated tumors. Imaging also shows liver accumulation while the kidney is also observed (Figures 7 and S4). Both the lipoplexes and ternary complexes therefore appear amenable to biodistribution enhancement by FUS hyperthermia such that tumor accumulation can be improved.

## Conclusions

In this study, we have formulated a novel siRNA delivery system comprising PEG-lipids, cationic lipids, and pH-responsive LAH4-L1 peptide that yields improved noncytotoxic, in vitro gene silencing. Substantial tumor delivery of siRNA using both lipoplexes and the new ternary complex is achieved with the aid of FUS-induced hyperthermia, and our findings confirm the positive impact of noninvasive focused ultrasound hyperthermia on siRNA complex tumor accumulation. FUS-induced tumor-enhanced uptake could offer a valuable tool to understand the effect of siRNA-increased local dose on treating tumors.

## Supporting Information Available

Encapsulation efficiency; calculated N:P ratio; siRNA encapsulation efficiency (Figure S1); complex stability (Figure S2); LAH4-L1 promotes siRNA lipid complex uptake in vitro (Figure S3); Biodistribution of excised organs after administration of complexes (Figure S4) (PDF)
