# Supplementary material for: Development of Cationic Lipid LAH4-L1 siRNA Complexes for Focused Ultrasound Enhanced Tumor Uptake
Source: Mol Pharm. 2023 Mar 29;20(5):2341–51. doi: 10.1021/acs.molpharmaceut.2c00909 (PMC10155207; doi:10.1021/acs.molpharmaceut.2c00909)
Supplement: Supplementary file 1 — mp2c00909_si_001.pdf [file mp2c00909_si_001.pdf]

Supplementary Material

Development of cationic lipid LAH4-L1 siRNA complexes for focused ultrasound enhanced tumour uptake

Shahd Abuhelal, Miguel N. Centelles, Michael Wright, A. James Mason, Maya Thanou*
Institute of Pharmaceutical Science, School of Cancer and Pharmaceutical Sciences, King's College London, Franklin-Wilkins Building, 150 Stamford Street, London, SE1 9NH, United Kingdom

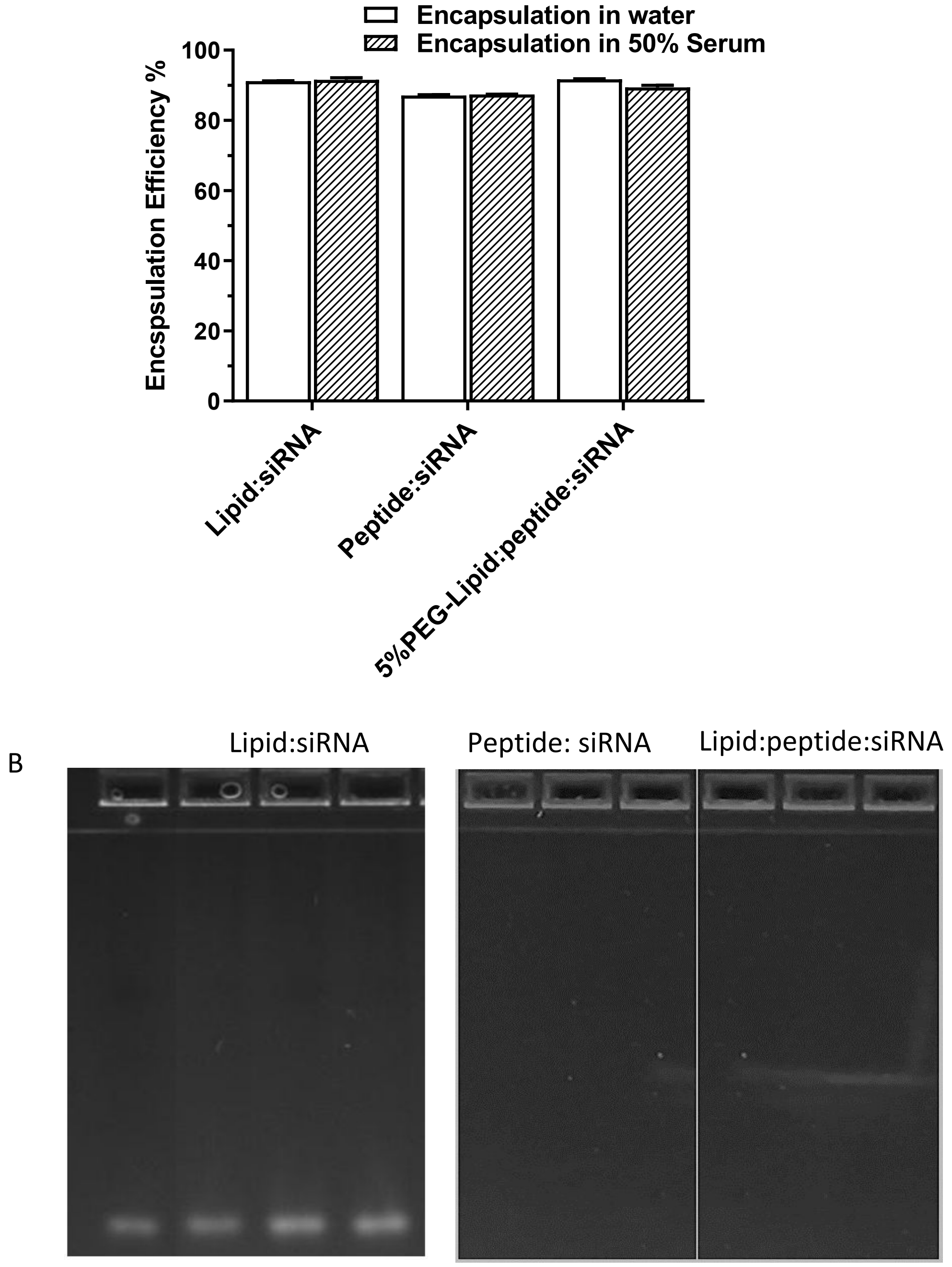

Figure S1: siRNA encapsulation efficiency of Lipid:siRNA complex (4:1 molar ratio), peptide:siRNA complex(10:1 w:w ratio), and Lipid:peptide:siRNA complex (4:9:1 w:w:w ratio), confirmed by A) Encapsulation efficacy using PicoGreen fluorometric assay after incubation in either water and/or 50% fetal bovine serum conditions. B) Agarose Gel electrophoresis of complexes after incubation with 4% Triton X-100 to release siRNA each column represents three replicate samples for complexed siRNA.

Calculated N:P ratio for Lipoplex and Ternary complex used in Figures 3-6

Lipid:siRNA: N:P = 5:1

Peptide:siRNA (10:1 wt:wt): N:P = 6.12 at neutral pH and 11.01 at acidic pH

Lipid:peptide:siRNA (4:9:1 wt:wt:wt): N:P = 5.77:1

Calculated N:P ratio for Ternary complexes screened in Figure 2.

Lipid:peptide:siRNA wt ratio	N:P Ratio
1:6:1	3.45:1
2:6:1	3.7:1
3:6:1	3.94:1
4:6:1	4.166:1
1:8:1	4.525:1
2:8:1	4.77:1
3:8:1	5.02:1
4:8:1	5.28:1
1:10:1	5.59:1
2:10:1	5.83:1
3:10:1	6.07:1
3.5:10:1	6.07:1
4:10:1	6.29:1
3.5:9:1	5.55:1
4:9:1	5.77:1

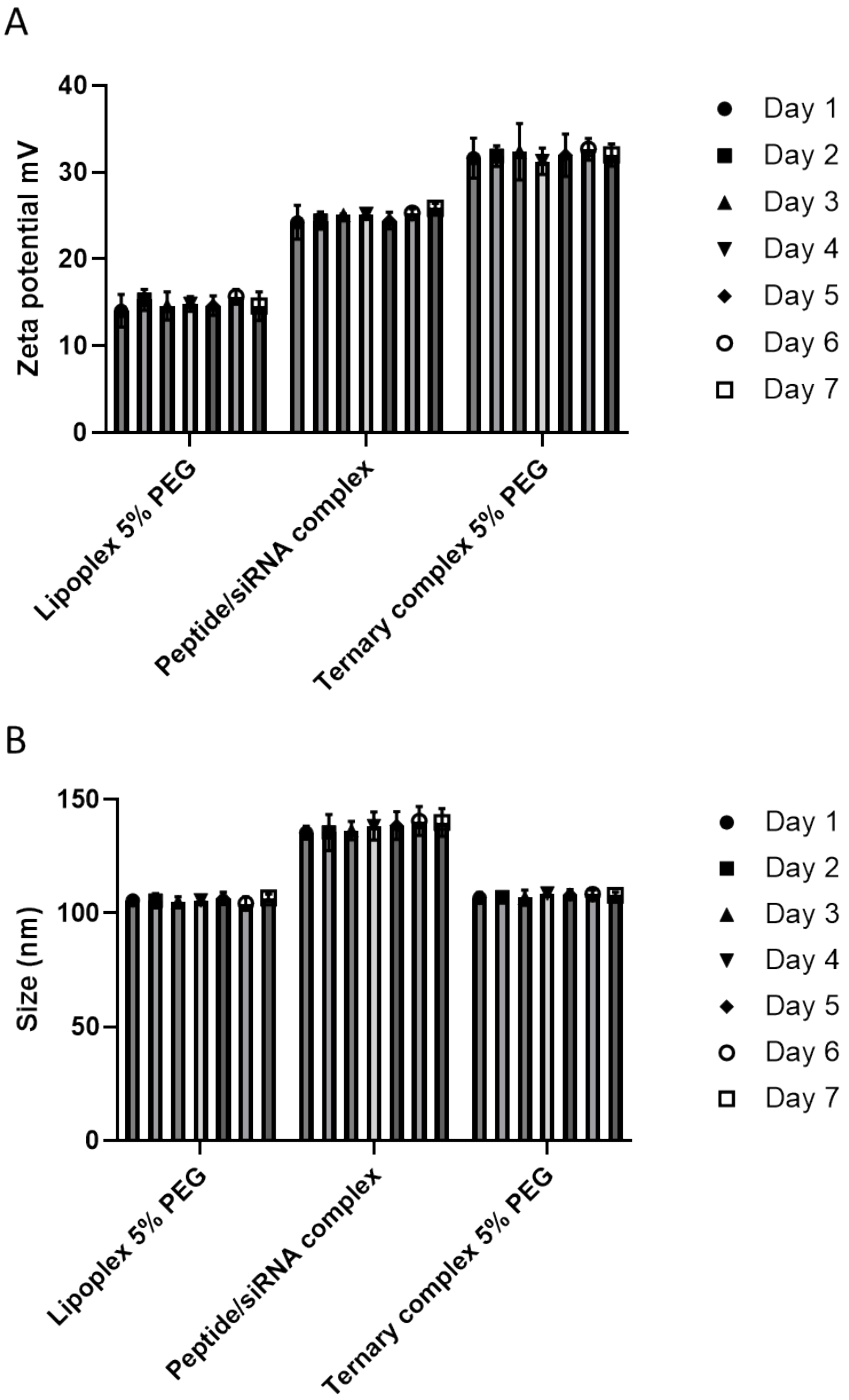

Figure S2. Complex stability. Complexes zeta potential (A) and size (B) stability over a week in 4 mM HEPES (pH 7.4) buffer. Data is mean ± SD.

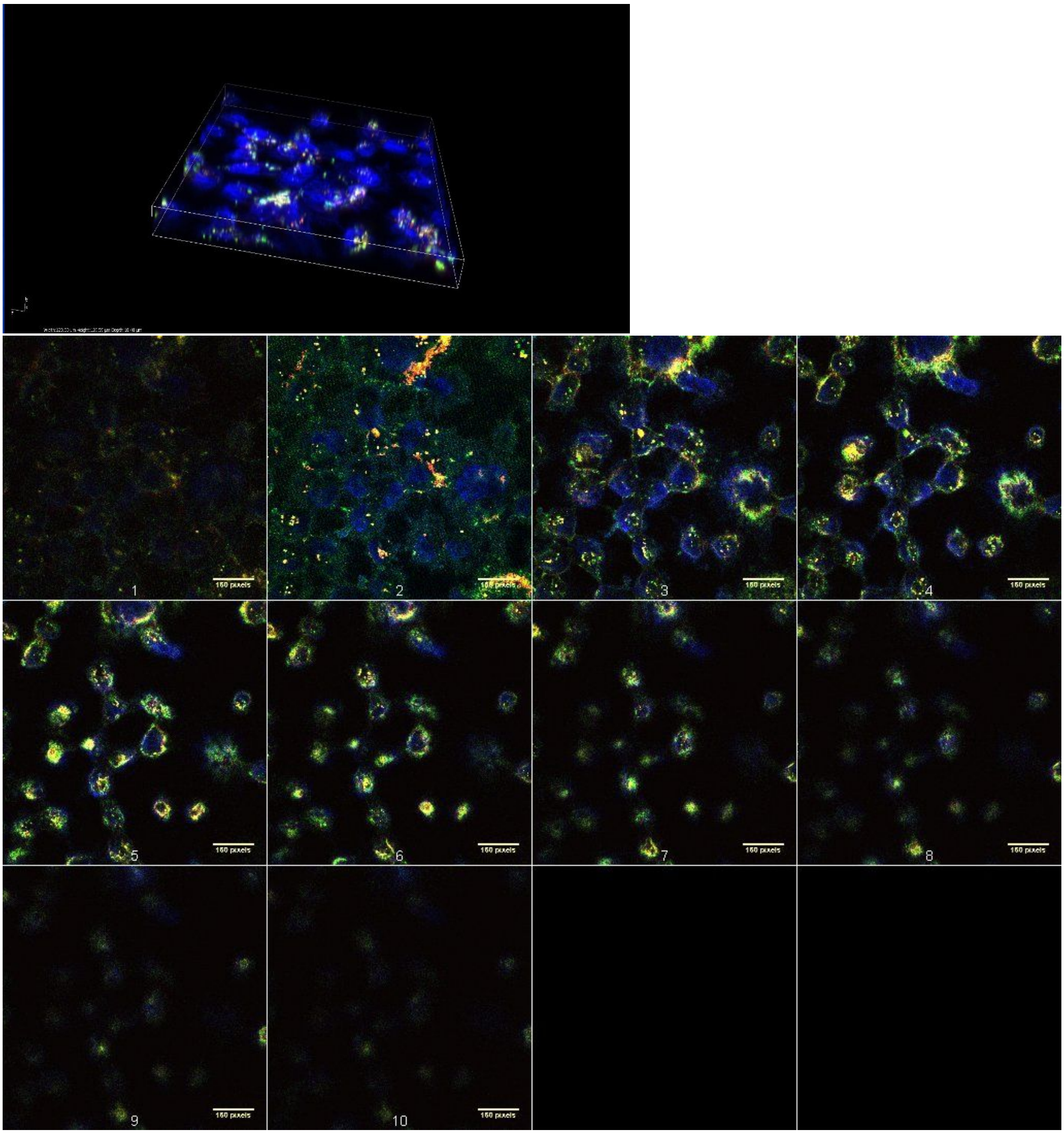

Figure S3 LAH4-L1 promotes siRNA lipid complex uptake in vitro even in the presence of PEGylated lipids. Upper panel. Upper panel: 3D reconstruction of Confocal Fluorescence microscopy cross sections of MDA-MB231 cells Lower panel: series of Z-sections used for the 3D image. Cells were treated with 70 nM FAM-siRNA (green) complexes containing Rhodamine-lipid (red) while cell nuclei are stained with DAPI (blue). Scale corresponds to 20 µm. Note the separate location of the lipid signal compared to siRNA signal indicating dissociation of the components within the cytosol.

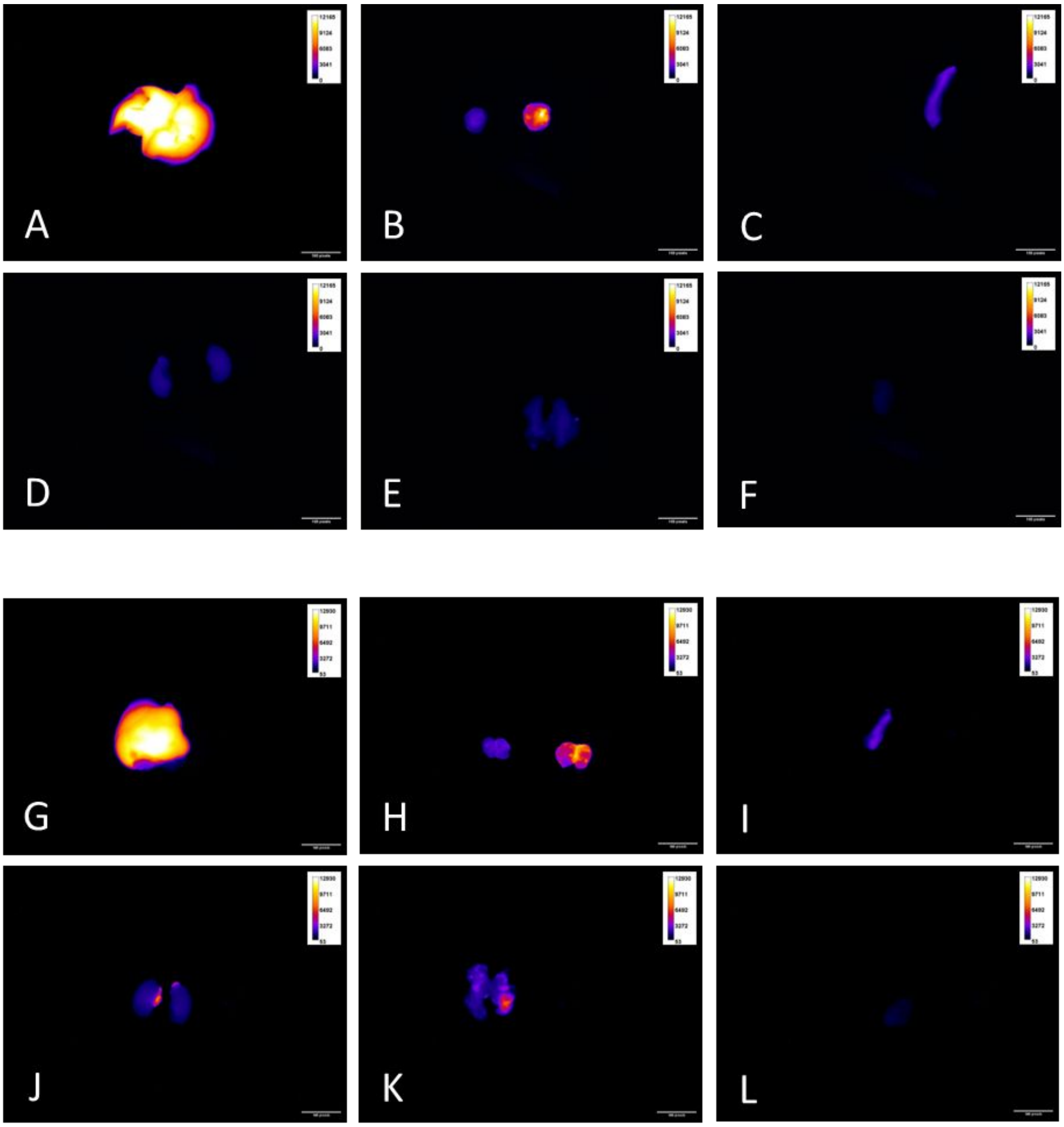

Figure S4: Biodistribution of excised organs after administration of complexes carrying near infrared fluorescently labelled siRNA. SHO mice bearing two tumours at either flank were administered 5% PEG lipid:siRNA (A-F) or 5% PEG lipid:peptide:siRNA (G-L) carrying Hylite®-labelled siRNA with the right flank tumour subjected to FUS. NIRF signal of: liver (A, G); left and right excised tumours (B, H); spleen (C, I); kidneys (D, J); lungs (E, K) and; heart (F, L) at 24h post treatment. Images representative of three (lipoplex) or two (ternary complex) mice.
